# Whole-Genome Expression Profiling in Skin Reveals SYK As a Key Regulator of Inflammation in Experimental Epidermolysis Bullosa Acquisita

**DOI:** 10.3389/fimmu.2018.00249

**Published:** 2018-02-15

**Authors:** Unni K. Samavedam, Nina Mitschker, Anika Kasprick, Katja Bieber, Enno Schmidt, Tamás Laskay, Andreas Recke, S. Goletz, Gestur Vidarsson, Franziska S. Schulze, Mikko Armbrust, Katharina Schulze Dieckhoff, Hendri H. Pas, Marcel F. Jonkman, Kathrin Kalies, Detlef Zillikens, Yask Gupta, Saleh M. Ibrahim, Ralf J. Ludwig

**Affiliations:** ^1^Department of Dermatology, University of Lübeck, Lübeck, Germany; ^2^Lübeck Institute of Experimental Dermatology, University of Lübeck, Lübeck, Germany; ^3^Institute for Medical Microbiology and Hygiene, University of Lübeck, Lübeck, Germany; ^4^Department of Experimental Hematology, Sanquin Research Institute, Amsterdam, Netherlands; ^5^Center for Blistering Diseases, Department of Dermatology, University Medical Center Groningen, University of Groningen, Groningen, Netherlands; ^6^Institute of Anatomy, University of Lübeck, Lübeck, Germany

**Keywords:** skin, autoimmunity, spleen tyrosine kinase, signal transduction, animal models, treatment, pemphigoid, epidermolysis bullosa acquisita

## Abstract

Because of the morbidity and limited therapeutic options of autoimmune diseases, there is a high, and thus far, unmet medical need for development of novel treatments. Pemphigoid diseases, such as epidermolysis bullosa acquisita (EBA), are prototypical autoimmune diseases that are caused by autoantibodies targeting structural proteins of the skin, leading to inflammation, mediated by myeloid cells. To identify novel treatment targets, we performed cutaneous genome-wide mRNA expression profiling in 190 outbred mice after EBA induction. Comparison of genome-wide mRNA expression profiles in diseased and healthy mice, and construction of a co-expression network identified *Sykb* (spleen tyrosine kinase, SYK) as a major hub gene. Aligned, pharmacological SYK inhibition protected mice from experimental EBA. Using lineage-specific SYK-deficient mice, we identified SYK expression on myeloid cells to be required to induce EBA. Within the predicted co-expression network, interactions of *Sykb* with several partners (e.g., *Tlr13, Jdp2*, and *Nfkbid*) were validated by curated databases. Additionally, novel gene interaction partners of SYK were experimentally validated. Collectively, our results identify SYK expression in myeloid cells as a requirement to promote inflammation in autoantibody-driven pathologies. This should encourage exploitation of SYK and SYK-regulated genes as potential therapeutic targets for EBA and potentially other autoantibody-mediated diseases.

## Introduction

In pemphigoid disease (PD), autoantibodies against defined structural proteins of the skin cause inflammation and subsequently subepidermal blistering (1, 2). During the last years, animal models of the PD epidermolysis bullosa acquisita (EBA) shaped our current understanding of the disease pathogenesis (3). In EBA, autoantibody-induced inflammation and subepidermal blistering are initiated by binding of autoantibodies to their target antigen [type VII collagen (COL7)]. Subsequently, the complement cascade is activated (4–6). This and cytokine release permit a CD18/ICAM1-dependent extravasation of Gr-1^+^ myeloid cells (7, 8). Within the skin, binding of myeloid cells to the immune complexes (ICs) located at the dermal–epidermal junction (DEJ) through specific Fc gamma receptors (FcγR) is unequivocally required to induce clinically manifest disease (9). The engagement of FcγR to skin-bound ICs triggers intracellular signaling, involving PI3K beta, AKT, p38 MAPK, ERK, Src family kinases, PDE4, CARD9, and RORα (10–15); ultimately leading to the release of reactive oxygen species (ROS) and proteases that facilitate inflammation and subepidermal blistering (7, 16).

To obtain further insights into EBA pathogenesis and to define novel treatment targets, we determined the whole-genome expression profile of 190 outbred mice of an advanced intercross line (17) after EBA induction. Of the identified hub genes, we functionally validated the spleen tyrosine kinase (*Sykb*, SYK). SYK is a non-receptor cytoplasmic enzyme that is mainly expressed in hematopoietic cells, which is essential in regulating cellular responses to extracellular antigens or antigen-immunoglobulin complexes (18, 19). As an important example, SYK acts downstream of activating FcγR and has thus emerged as a drug target for antibody-induced diseases, such as rheumatoid arthritis, where autoantibody-induced inflammation depends on FcγR. Yet, we recently described anti-inflammatory properties of SYK, triggered by binding of highly galactosylated ICs to FcγRIIB and dectin-1 to block the pro-inflammatory signaling triggered by G-protein coupled, membrane-bound receptors, exemplified by the C5aR1. The inhibition of signaling downstream of the C5aR1 was mediated by tyrosine phosphorylation of the ITAM-like motif downstream of dectin-1 and transient phosphorylation of SYK (6). Thus, inhibition of SYK may have either anti- or pro-inflammatory effects.

Hence, to address the functional role of the identified hub gene *Sykb* in experimental EBA, we performed an in-depth functional analysis using a variety of *in vitro* and *in vivo* model systems. Ultimately, we also verified the predicted gene network of the *Sykb* hub gene, leading to the identification of novel *Sykb*-interacting genes.

## Materials and Methods

### Experiments with Human Biomaterials

Foreskin and blood collections from healthy volunteers and patients were performed after written informed consent was obtained. All experiments with human samples were approved by the ethical committee of the Medical Faculty of the University of Lübeck and were performed in accordance with the Declaration of Helsinki. Skin biopsies from bullous pemphigoid (BP) patients for RNA expression profiling were obtained from five patients. The diagnosis was based on clinical presentation, detection of linear IgG and/or C3 deposits along the DEJ in perilesional skin biopsies by direct IF microscopy, and the detection of circulating anti-NC16A IgG by ELISA. Biopsies for RNA expression profiling were obtained from perilesional skin biopsies. Biopsies from corresponding, non-affected body sites served as controls. We included four female and one male BP patient. The mean age was 87 years (range 83–90 years). RNA expression profiling was performed using the Human Gene 1.0ST (Affymetrix, Santa Clara, CA, USA). The complete dataset is currently under analysis. Herein, we focused the analysis on *SKY* and contrasted the expression levels of perilesional versus control skin. The data (CEL files) were processed using R oligo package. The data were corrected for background, and RMA normalization was performed. The normalized gene expression for *SYK* was used for accessing statistical significance.

### Animal Experiments and Immunization-Induced Murine EBA

C57Bl/6 (B6) mice were obtained from Charles River Laboratories (Sulzfeld, Germany). Mice expressing the cre gene on LysM [B6.129P2-Lyz2tm1(cre)Ifo/J], CD2 [B6.Cg-Tg(CD2-cre)4Kio/J], and SYK-loxP-flanked (B6.129P2-Syktm1.2Tara/J) strains were obtained from the Jackson Laboratory (Bar Harbor, ME, USA) and were crossed to obtain cell lineage-specific Syk deletions. Mice were housed under specific pathogen-free conditions and provided standard mouse chow and acidified drinking water *ad libitum*. Animal experiments were approved by local authorities of the Animal Care and Use Committee (Kiel, Germany) and performed by certified personnel. Skin specimens from mice with immunization-induced EBA were obtained for unbiased mRNA expression profiling (see below) from a previously published study (17) using mice of an autoimmune-prone intercross outbred line (AIL mice). In brief, in order to generate a genetically diverse mouse line, EBA-susceptible (MRL/MpJ) and -resistant mice (NZM2410/J, Cast, and BXD2J), and the offspring of each generation were intercrossed for several generations. Genetic diversity of this so termed four-way, autoimmune-prone intercross mouse line, is reflected by the different morphological traits, including weight, tail length, or fur color. For the induction of experimental EBA, mice of the fourth generation were used.

### Generation of Microarray Data (miRNAs/Genes) and Bioinformatics Analysis

To monitor gene expression in the skin samples derived from AIL mice, total RNA was extracted from ears and hybridized to the Affymetrix Mouse Gene 1.0 ST Array (Affymetrix, Santa Clara, CA, USA). Raw data were processed using the “oligo” R package. The RMA method incorporated in the R package was used for normalization of probe intensities for all samples (20). The limma R package was used to assess differentially expressed genes between the EBA and wild-type mice. *p*-Values were corrected for multiple testing using Bonferroni correction. For gene expression profiling, low intensity probes were omitted to avoid putative false-positive signals and to lower the barrier for correction of multiple testing; these probes were filtered out using the median-based method as implemented in the function “expressionBasedfilter” from the R DCGL package (21). Additionally, genes that did not show significant variations (*p*-value < 0.05) across the samples were filtered using the function “varianceBasedfilter” in the same package. This function reduces the data to the most variable genes, which are presumably critical for the phenotype.

### *Ab Initio* Gene and Network Predictions

For *ab initio* prediction, genes were clustered based on their co-expression profiles across different samples. The standard weighted correlation network (WGCNA) analysis procedure was used for cluster or module detection (22). A weighted adjacency matrix of pair-wise connection strengths (correlation coefficients of gene expression levels) was constructed using the soft-threshold approach with a scale-independent topological power β = 6 (mRNA). Scale-free topology is a network whose degree of distribution follows a power law. For each probe, the connectivity was defined as the sum of all connection strengths with all others. Probes were aggregated into modules by hierarchical clustering and refined with the dynamic cut tree algorithm (23). Pearson correlation coefficient was determined for each phenotype-module pair. The representative module expression profiles or module eigengene values are the first principal component of the gene expression profiles within a module. The correlation between the module eigengene and the sample trait of interest yields the eigengene significance as assessed by a correlation test. Modules were assigned different colors, with gray assigned to traits that could not be clustered in any other module. Additionally, partial least squares (PLS) regression was used to filter out false-positive interactions among the genes in each module (24). Briefly, PLS measures associations between each pair of genes under the influence of all other genes present in the dataset. Thus, it assigns the weight or numerical measurement for each edge/interaction for each pair of genes. The statistical significance of these edges is calculated using an empirical Bayes technique that uses a false discovery rate to assess significance (25). The DAVID web software was used for pathway and gene ontology analysis (26). The software is based on the modified Fisher’s exact test and performs gene ontology analyses for the group of genes based on metabolic pathways, cellular components, and biological functions. Briefly, DAVID has pre-defined database in which various sets of genes are assigned to ontology terms such as metabolic pathways, cellular compartments, and biological processes. DAVID provides a list of genes. Thereafter, DAVID calculates how many genes in the list are associated with a specific ontology term. The significance of the ontology term for these genes is calculated by the modified Fisher’s exact test. *p*-Values obtained for each ontology term are corrected for multiple testing using Bonferroni corrections.

### IC-Induced Activation of Polymorphonuclear Leukocytes (PMNs)

Anti-coagulated (EDTA) blood collected from healthy blood donors was used for PMN isolation. Inhibition of ROS release in presence of BAY 61-3606 or PRT062607 (Selleckchem, Munich, Germany) was measured from IC- or C5a-activated PMNs using published protocols (27, 28).

### IC-Induced Neutrophil Activation

Peripheral heparinized blood was collected by venipuncture from healthy adult volunteers. Neutrophils were isolated as described previously using Percoll gradient centrifugation (29). Plate-bound immobilized immune complexes (iICs) were formed using a human serum albumin (HSA) antigen and a rabbit polyclonal anti-HSA-IgG1 antibody as described (30). Neutrophil activation was assayed by ROS release as described earlier.

### Determination of Cell Surface Marker Expression

Activation of PMN in the presence of BAY61-3606 was assayed using flow cytometry following established protocols (11, 31).

### Measurement of PMN-Dependent Dermal–Epidermal Separation

*Ex vivo* induction of dermal–epidermal separation was performed as previously described (32). In brief, cryosections of human neonatal foreskin were incubated with recombinant anti-COL7 antibodies (32). After washing, slides were incubated for 2 h at 37°C with PMN, isolated by dextran sedimentation of freshly collected, heparinized blood from healthy volunteers. Here, sections were treated with either solvent or BAY61-3606 at the indicated concentrations. After fixation in 4% formaldehyde, slides were stained by H&E and examined by light microscopy for the presence of split formation within the DEJ zone. An observer unaware of the sections treatment has performed the later step. The degree of dermal–epidermal separation is expressed in relation to the length of the entire DEJ zone of each section. A detailed, step-by-step protocol for this assay has recently been published (33).

### Evaluation of PMN Viability

The viability of PMN in presence of BAY61-3606 was assayed using the FITC Annexin V Apoptosis Detection Kit II following the protocol provided by the manufacturer (BD Pharmigen™, Heidelberg, Germany). PMNs treated with UV light for 20 min served as positive control.

### Activation of Neutrophils by iICs and Western Blotting Analysis

Neutrophils (5 × 10^6^ in 1 ml of RPMI 1640 containing 10% heat-inactivated FCS) were pre-incubated in presence or absence of 250 ng/ml of the Syk BAY 61-3606 (medchemexpress, Princeton, NJ, USA) inhibitor for 20 min at 37°C. Subsequently, cells were added to wells coated with HSA-anti-HSA iIC and incubated for 15 min at 37°C. Following iIC stimulation, whole-cell lysates were prepared using TCA as previously described (34). Western blotting analysis was performed using Abs against human phospho-Akt (Thr308), phospho-p44/42 MAPK (ERK1/2 and Thr202/Tyr204), phospho-p38 MAPK (Thr180/Tyr182), and β-actin (all from Cell Signaling Technology) and probed with HRP-conjugated anti-rabbit or anti-mouse IgG (New England Biolabs, Beverly, MA, USA).

### Anti-COL7 IgG Transfer-Induced Murine EBA and Treatment Protocol

To induce experimental EBA, mice were injected on alternating days with anti-COL7 IgG according to established protocols (35). In brief, New Zealand white rabbits were s.c. immunized with 250 mg murine von Willebrand factor A-like domain 2 protein that was suspended in CFA. The animals were boosted three times (at 13-day intervals) with the same protein preparation in IFA, and immune sera were characterized using immunofluorescent (IF) microscopy on cryosections of murine skin. IgG from immune and normal rabbit sera was purified using protein G affinity. Immune rabbit IgG or normal rabbit IgG (5 mg/injection) was injected s.c. into adult mice every second day for a total of six injections. Some mice were treated with BAY61-3606 dissolved in water. Mice were treated twice-daily p.o. with either solvent or BAY61-3606 at a dose of 25 or 50 mg/kg body weight. Treatments were initiated 1 day prior to the first anti-COL7 IgG injection, applied daily, and maintained until day 11. Clinical disease manifestation (expressed as the percentage of body surface area covered by EBA skin lesions) was determined 4, 8, and 12 days after the initial anti-COL7 IgG injection. From these data, the area under the curve (AUC) was used to calculate overall EBA severity.

### Immunofluorescence Microscopy

Biopsies of non-lesional skin were obtained 2 days after the last IgG injection, and IgG and C3 deposits were detected by direct immunofluorescence (IF) microscopy. The sections were probed with 100-fold diluted fluorescein isothiocyanate (FITC)-labeled antibodies specific to rabbit IgG (Dako, Glostrup, Denmark and Abcam plc, Cambridge, UK) and FITC-labeled anti-murine C3 (MP Biomedicals, Solon, OH, USA).

### Flow Cytometry

For FACS analysis, healthy and lesional skin (both from corresponding anatomical sites) or blood was taken from mice after induction of experimental EBA. Single cell solutions from and blood were erythrocyte lysed with RB cell lysis buffer (Miltenyi). The skin samples were cut into small pieces and digested with 345 mg/ml liberase (Roche) in RPMI for 30 min/37°C. Single cells were stained for the following surface markers using standard FACS procedures: CD45-VioGreen (clone: 30F11), CD3-VioBlue (clone: 17A2), Ly6C-FITC (clone: 1G7.G10), as well as Ly6G-APC Vio770 (clone: 1A8) and for blood CD19-APC (clone: 6D5), all from Miltenyi. For the subsequent intracellular staining, the cells were fixed in fixation buffer (BioLegend) and permeabilized using the Intracellular Staining Perm Wash Buffer (BioLegend) following the manufacturer’s protocol. Intracellular staining was performed with SYK-PE (clone: 4D10.2). Cells were first gated for scatter (SSC-A/FSC-A) and singlets (FSC-H/FSC-A). The CD45^+^ gates were further analyzed for double-positive staining of SYK with the appropriate cell markers. Measurements were performed at the Miltenyi MacsQuant10, and data were analyzed with the MACSQuantify™ Software (version 2.8).

### RT-PCR

For gene expression analysis, TaqMan gene expression assays were purchased for the following transcripts: Plaur Mm01149438_m1, formyl peptide receptor 1 (Fpr1) Mm00442803_s1, CD3001b Mm01701741_m1, and *Gapdh* Mm99999915_g1 (Thermo Fisher Scientific, Waltham, MA, USA). RNA isolation, reverse transcription, and real-time RT-PCR were performed as described (36). All data were normalized to *Gapdh*.

### Western Blot Analysis

Sections of frozen skin tissue (20 μm × 20 μm, either skin from immunization-induced EBA or corresponding healthy skin sections from Titermax™-injected controls) were scraped using a 30 G syringe in 100 µl RIPA buffer and the protein concentration was measured using BCA protein assay following manufacturer’s protocols (Thermo Fisher Scientific). 15 µg protein were mixed in 5× Laemmli buffer and heated for 5 min at 95°C. Samples were separated by 10% SDS-PAGE and transferred to Immobilon-P membrane (Millipore, Bedford, MA, USA). The membrane was blocked for 1 h with TBS containing 0.1% Tween 20 and 5% skim milk or 5% BSA and incubated for overnight (4°C) with the primary antibody (either SYK, clone D3Z1E (XP) or Rabbit mAb (Cell Signaling Technology, Danvers, MA, USA)) diluted in blocking buffer following the manufacturer’s instructions. Then the membrane was washed three times for 5 min with TBS containing 0.1% Tween 20 and incubated with peroxidase-conjugated anti-rabbit secondary antibodies (Dako Deutschland GmbH, Hamburg, Germany) for 1 h at room temperature. After a washing step, the membrane was incubated for 1 min with ECL reagent (GE Healthcare Europe GmbH, Freiburg, Germany) and exposed to film. For reprobing with GAPDH antibody (Cell Signaling Technology), the membrane was washed twice in PBS, stripped for 10 min/37°C with stripping buffer (GE Healthcare Europe GmbH), and washed three times for 5 min with TBS at room temperature. The relative SYK expression was calculated using ImageJ 1.51f (NIH, USA), and after background subtraction, the relative mean density of SYK/GAPDH was calculated.

### Histopathology

Skin sections from corresponding anatomical sites were obtained 2 days after the last IgG injection and prepared for examination by histopathology as described. The dermal neutrophil cell infiltrate was assessed semi-quantitatively using a score ranging from 0 to 3 indicating no, mild, moderate, or severe infiltration, respectively (37).

### Statistical Analysis

Unless otherwise noted, data were presented as mean ± SD. For comparisons of two groups, *t*-test or Mann–Whitney Rank Sum test was used when appropriate. For comparisons of more than two groups, ANOVA was used. For equally distributed data, one-way ANOVA followed by Bonferroni *t*-test for multiple comparisons was used; if the data were non-parametric, ANOVA on ranks (Kruskal–Wallis) was applied followed by Bonferroni *t*-test for multiple comparisons. In all tests, *p* < 0.05 was considered significant. All statistical analyses were performed using SigmaPlot 13.0 (Systat Software, Erkrath, Germany). The number of replicates for each experiment is detailed at the respective table/figure legends.

## Results

### Cutaneous mRNA Expression Profiling Identifies SYK As One of the Major Differently Expressed Hub Genes in Experimental EBA

**Figure 1:**
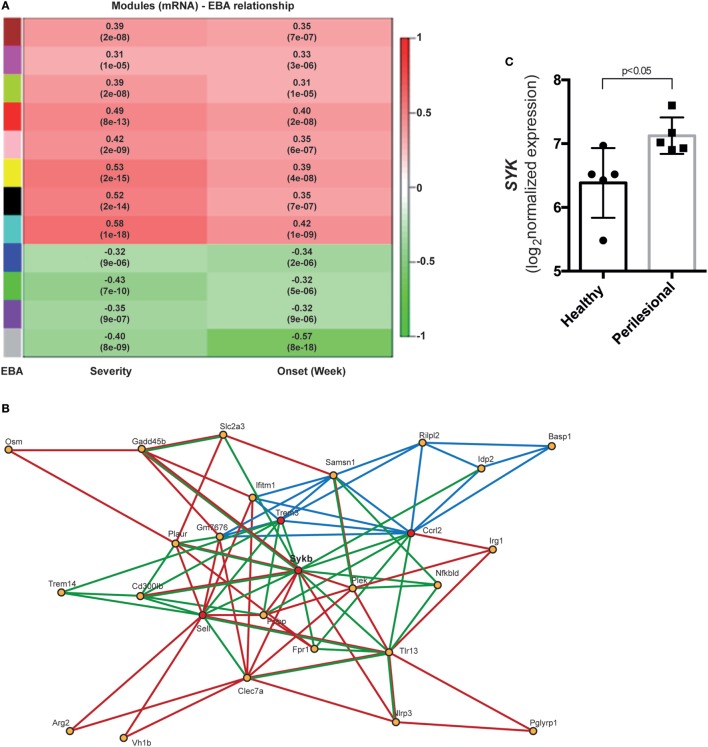
Differential co-expression of cutaneous mRNA expression levels in mice with experimental epidermolysis bullosa acquisita (EBA) identifies SYK as a major hub gene candidate. **(A)** We applied hierarchical clustering to cluster gene profiles and the dynamic cut tree method to combine branches and define co-expressed groups (modules) from the expression data. We found 12 modules and assigned them different colors, with gray used for genes that could not be assigned to any other module. To identify disease-related modules, we correlated module eigenvalues to the disease score for EBA severity and onset. Bar on the left corresponds to modules, and bar on the right displays the range for Pearson correlation (from −1 to 1). Data are derived from 68 mice with experimental EBA and 122 mice that remained healthy after type VII collagen immunization (17). **(B)** This image shows the gene network of the yellow module. Red lines display the interaction information from STRING database. Green lines display predicted interactions using domain interaction information. Dark blue lines represent PLSR-based interactions. Red circles indicate hub genes. **(C)** In patients with bullous pemphigoid, which shares a lot of similarity with the inflammatory EBA variant (2), we observed an increased SYK expression in perilesional, as compared to non-affected skin of corresponding anatomical sites.

From mice of the outbred mouse line, 68 had clinically manifest EBA, while the remaining 122 mice were clinically healthy. Collectively, 1,038 mRNA probes were differentially expressed (adjusted *p* < 0.05, Bonferroni corrected). Of these, in samples from mice with clinically manifest EBA, 425 probes were downregulated and 613 were upregulated (Tables S1 and S2 in Supplementary Material). Next, a gene co-expression network of differentially expressed genes was constructed. Of the identified 12 modules, 8 showed a positive correlation, while 4 negatively correlated with the EBA phenotype (Figure 1A). In the yellow module (*r* = 0.53, *p*-value = 2e−15, Figure 1B), 86 interactions among the genes were validated by different statistical and database approaches (DOMINE, STRING, IPA databases, and PLS regression). Furthermore, four hub genes (degree of interaction > 10) were identified: *Sykb, Ccrl2, Sell*, and *Trem3*. We focused on the yellow module, specifically, *Sykb* because it has emerged as a therapeutic target in autoimmune diseases (38), and we observed an increased expression of *SYK* mRNA in perilesional skin of BP patients (Figure 1C). Our interest in SYK was further provoked by the contrasting effects of its blockade in mouse models and clinical trials in arthritis (39, 40).

### Pharmacological SYK Blockade Dose-Dependently and Almost Completely Impairs Induction of Experimental EBA in Mice Induced by Transfer of Anti-COL7 IgG

**Figure 2:**
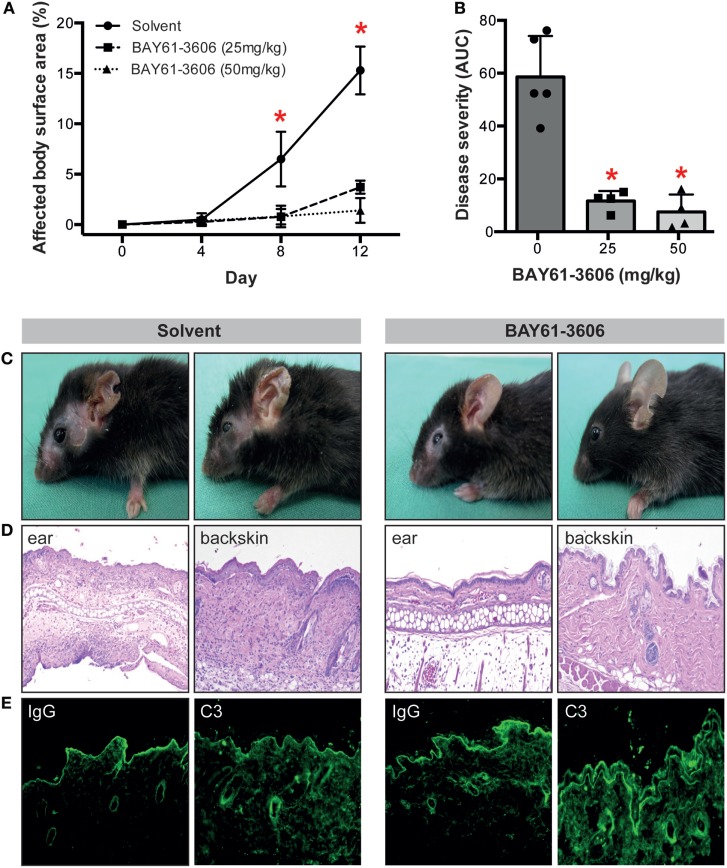
The SYK inhibitor BAY61-3606 protects mice from inflammation in antibody transfer-induced epidermolysis bullosa acquisita (EBA). **(A)** Treatment of mice with the SYK inhibitor BAY61-3606 almost completely protected them from induction of experimentally induced EBA by transfer of anti-type VII collagen IgG. Graph shows the mean (SD) body surface area affected by EBA skin lesions. **(B)** Overall disease activity was calculated as area under the curve (AUC) derived from individual mice in each of the treatment groups. Here, the mean (SD) of the AUC is shown. Individual dots represent disease severity (AUC) of individual mice (*p < 0.05, ANOVA with a Bonferroni post-test, *n* = 4–5 mice/group). Representative **(C)** clinical images of mice at day 12 of the experiment, **(D)** H&E-stained skin sections at 100× original magnification and **(E)** direct immunofluorescent microscopy staining for IgG and C3 from perilesional skin from solvent (left)- and BAY61-3606 (right)-treated animals (50 mg/kg dose) at 100× original magnification. For panels **(C,D)**, data are based on five solvent and four (per concentration) BAY61-3606 treated animals.

To validate the importance of SYK in EBA pathogenesis *in vivo*, we induced EBA in mice by anti-COL7 IgG transfer in the absence or presence of the SYK inhibitor BAY61-3606. Pharmacological blockade of SYK almost completely prevented the EBA-inducing activity of anti-COL7 IgG (Figures 2A–C). Consistent with the clinical observations, a reduction of dermal infiltration was observed in BAY61-3606-treated animals compared to the corresponding anatomical sites in controls (Figure 2D). These changes were independent of alterations in IgG or C3 deposition along the DEJ (Figure 2E).

### Induction of Anti-COL7 IgG-Induced EBA Requires SYK Expression in Cells of Myeloid, but Not Lymphoid Linage

**Figure 3:**
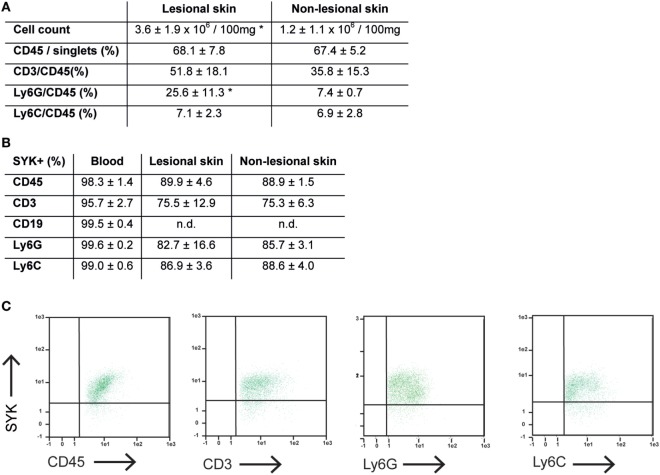
Expression of CD45^+^ cells is increased, while proportional expression of SYK in leukocytes remains constant in lesional versus non-lesional skin in mice with experimental epidermolysis bullosa acquisita (EBA). **(A)** Specimen from lesional and non-lesional skin of mice injected with anti-type VII collagen IgG were analyzed for quality and quantity of dermal leukocyte infiltration. Yield of cells was higher from lesional skin compared to non-lesional skin from corresponding sites. Regarding the relative distribution of all leukocytes (CD45^+^) or subsets, we observed a difference regarding neutrophils (CD45^+^/Ly6G^+^; *p < 0.05, *t*-test). Hence, the relative distribution of the dermal infiltrate remains constant, while the absolute number of CD45^+^ cell increases in the skin of mice with experimental EBA. CD19^+^ cells were not observed in the skin samples (not shown). **(B)** Separately, expression of SYK was evaluated in CD45^+^ cells obtained from lesional and non-lesional skin. Relative expression of SYK in CD45^+^, CD45^+^/CD3^+^, CD45^+^/Ly6G^+^, and CD45^+^/Ly6C^+^ was identical in both groups. CD45^+^ cells from blood were used as positive control. **(C)** Representative FACS stainings from lesional skin, gated on CD45^+^/singlet cells. All data are based on four to five samples per group.

We next aimed to identify the cellular source of SYK. Because SYK is mainly expressed in hematopoietic cells (18), we focused on these. We first evaluated the cellular composition within the dermal infiltrate of experimental EBA, and simultaneously determined if lymphoid and/or myeloid cells within the infiltrate express SYK. Comparison of lesional versus non-lesional skin of mice injected with anti-COL7 IgG at the end of the experiment showed a significant increase in cell numbers, while the proportion of most leukocytes within the dermis remained constant, the amount of Ly6G^+^ cells, which are key effector cells in experimental EBA, increased significantly (Figure 3A). In line with previous observations (41), all CD45^+^ cells expressed SYK, which, in addition, showed an identical proportional expression level in lesional and non-lesional skin (Figures 3B,C).

**Figure 4:**
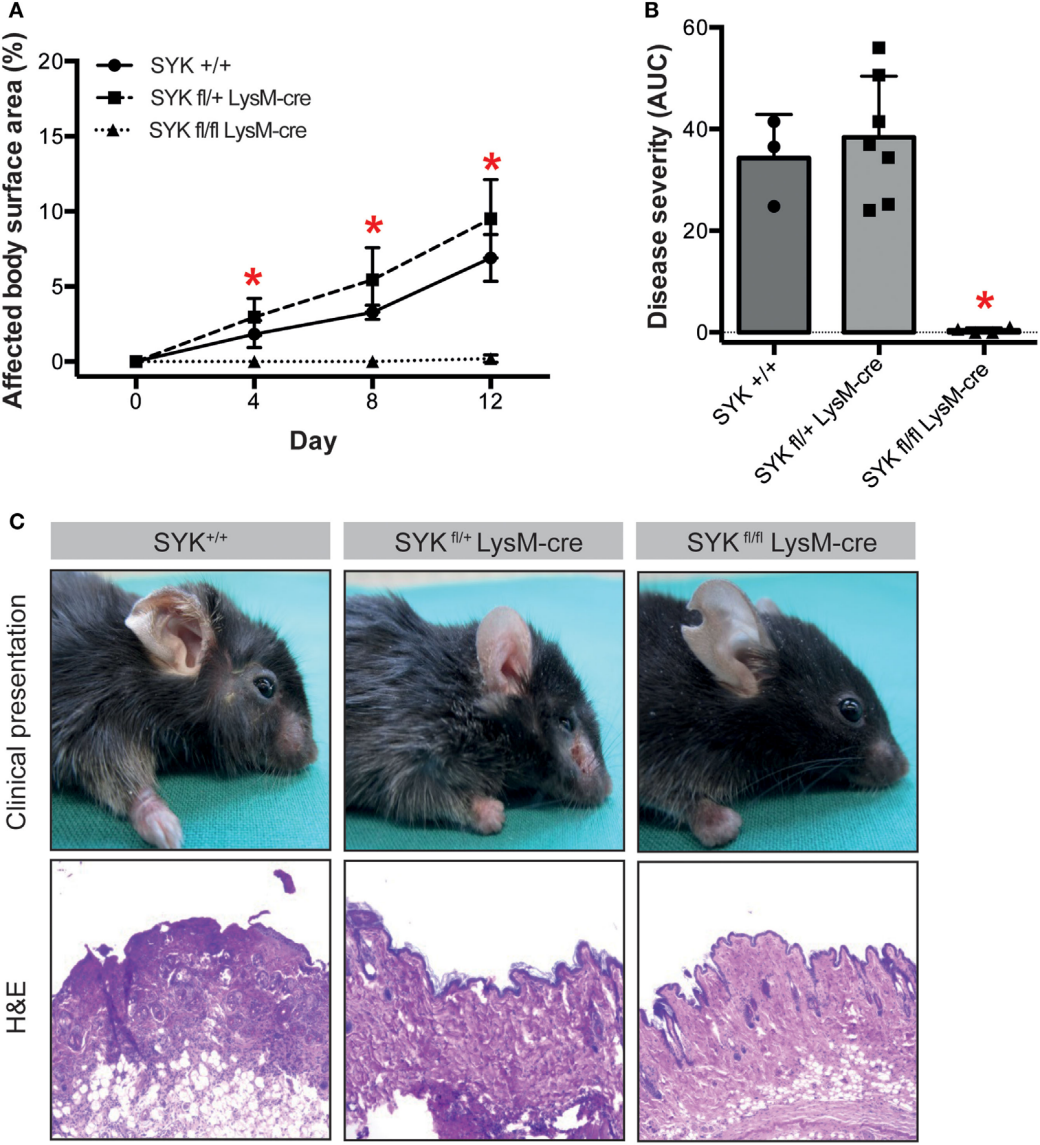
SYK^fl/fl^ LysM-Cre mice are completely protected from induction of antibody transfer-induced epidermolysis bullosa acquisita (EBA). **(A)** Mean (SD) of affected body surface area after repetitive injections of anti-type VII collagen IgG into indicated mouse strains over the 12-day observation period (*n* = 3–7/strain). SYK wild type (+/+) and mice with only one SYK allele in their myeloid cells (SYK^fl/+^ LysM-cre) developed clinically manifested skin lesions, whereas mice deficient for SYK in myeloid cells (SYK^fl/fl^ LysM-cre) were completely protected from induction of EBA (*p < 0.05, ANOVA with Bonferroni post-test). **(B)** The cumulative disease severity [area under the curve (AUC)] confirms complete protection of the SYK^fl/fl^ LysM-cre mice (*p < 0.05, ANOVA with a Bonferroni post-test). Individual dots correspond to the AUCs of single mice. **(C)** Representative clinical images and H&E-stained sections from the back skin of indicated mouse strains (100× original magnification). All images shown are from day 12 of the experiment.

**Figure 5:**
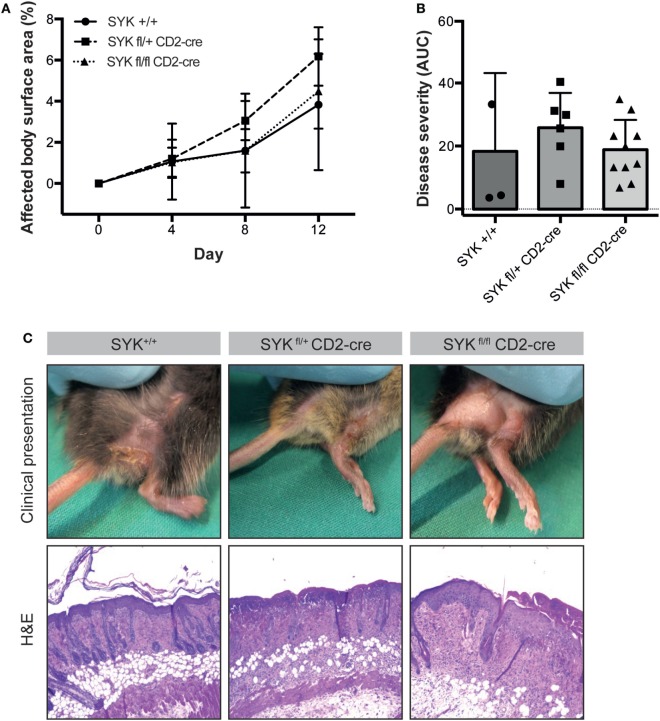
SYK^fl/fl^ CD2-Cre mice develop blistering in antibody transfer-induced epidermolysis bullosa acquisita (EBA). **(A)** Mean (SD) of the affected body surface area after repetitive injections of anti-type VII collagen IgG into the indicated mouse strains over the 12-day observation period (*n* = 3–9/strain). In contrast to the SYK^fl/fl^ LysM-cre mice, mice with the specific deletion of SYK in lymphoid cells were completely susceptible to EBA induction. **(B)** The cumulative disease severity [expressed as the area under the curve (AUC)] calculated from the graphs in panel **(A)** is shown. The individual dots correspond to the AUCs of individual mice. **(C)** Representative clinical images and H&E-stained sections from the back skin of the indicated mouse strains (100× original magnification). All images shown are from day 12 of the experiment.

To evaluate the functional impact of SYK expression on cells from the myeloid and lymphoid linage, floxed SYK mice were crossed with either LysM-Cre or CD2-Cre mice to selectively deplete SYK from myeloid or lymphoid cells. Notably, SYK^fl/fl^ LysM-Cre mice were completely protected from EBA induction by anti-COL7 IgG transfer (Figures 4A–C); while transfer of anti-COL7 IgG-induced experimental EBA in SYK^fl/fl^ CD2-Cre mice comparable to controls (Figures 5A–C). Changes in SYK^fl/fl^ LysM-Cre mice were independent of changes in IgG or C3 deposition along the DEJ. Overall, this indicated that SYK expression in myeloid cells is an absolute requirement for induction of experimental EBA.

### The SYK Inhibitor BAY61-3606 Dose-Dependently Blocks IC-Induced PMN Activation

**Figure 6:**
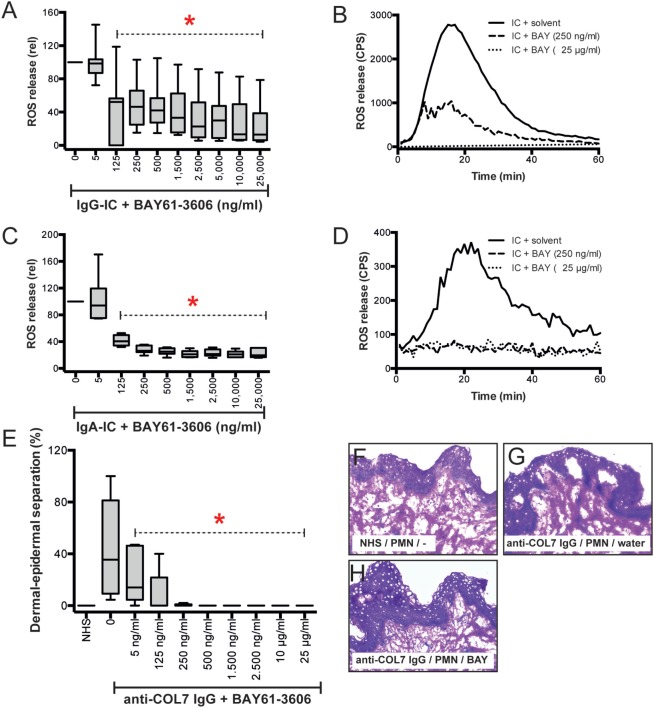

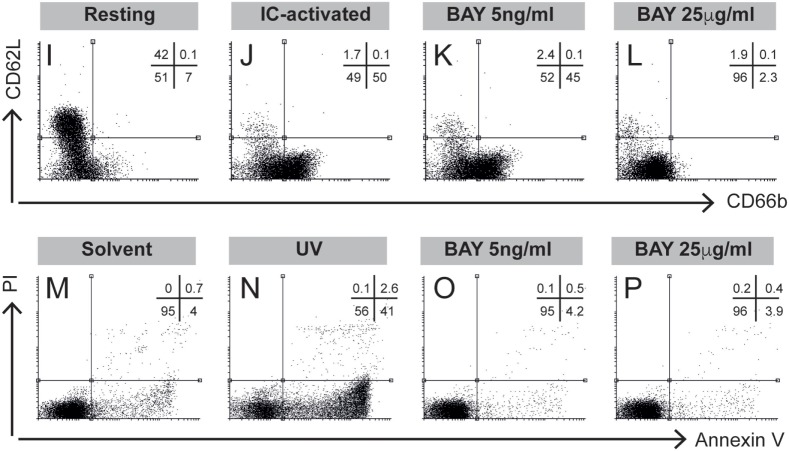
Pharmacological inhibition of SYK blocks immune complex (IC)-induced activation of polymorphonuclear leukocytes (PMNs) *in vitro* and *ex vivo*. **(A)** Human PMNs were activated using IgG-ICs, and their activation was determined by measuring the release of reactive oxygen species (ROS). The data (*n* = 11–30/group) were normalized to IC-activated PMNs in the presence of solvent (water) and are displayed as the median (black line), the 27/75 percentiles (box), and the 5/95 percentiles (error bars). BAY61-3606 reduced the release of ROS from IC-activated PMNs in a dose-dependent manner (ANOVA on Ranks with Dunn’s post-test). **(B)** Representative ROS release expressed as counts per second (CPS) over the 60-min experimental period. **(C)** Human PMNs were activated using IgA-ICs, and their activation was determined by measuring the release of ROS. The data (*n* = 6/group) were normalized to the IC-activated PMNs in the presence of solvent (water) and are displayed as the median (black line), the 27/75 percentiles (box), and the 5/95 percentiles (error bars). BAY61-3606 reduced the release of ROS from IC-activated PMNs in a dose-dependent manner (ANOVA on Ranks with Dunn’s post-test). **(D)** Representative ROS release expressed as CPS over the 60 min experiment. **(E)** BAY61-3606 also ablates dermal–epidermal separation in cryosections of human skin incubated with anti-type VII collagen (COL7) IgG and PMNs. The data are presented as the mean (boxes) and STD (error bars) and are based on five experiments per group. To calculate whether the effects of BAY61-3606 were significant, ANOVA with Ranks and Dunn’s post-test was used. **(F–H)** Representative images of the cryosection assay at a 200× original magnification showing **(F)** no dermal–epidermal separation in the sections incubated with normal human serum (NHS) and PMNs, **(G)** no dermal–epidermal separation in the sections incubated with anti-COL7 IgG, and **(H)** no dermal–epidermal separation in the sections incubated with anti-COL7 IgG, PMNs, and 25 mg/ml BAY61-3606. **(I–L)** Representative experiments evaluating the expression of CD66b (*x*-axis) and L-selectin (CD62L, *y*-axis) in immune complex-stimulated PMNs. **(I)** CD66b and CD62L expression in resting PMNs show low expression of CD66b and high expression of CD62L. Data are based on five experiments per group. **(J)** By contrast, IC activation leads to L-selectin shedding and increased CD66b expression. **(K)** Low concentrations of BAY61-3606 had no impact on the IC-induced changes in PMN surface molecule expression. **(L)** Higher compound concentrations normalized CD66b expression, but had no effect on L-selectin sheading. **(M–P)** These effects of BAY61-3606 were achieved at non-toxic concentrations, as evaluated by annexin V/propidium iodine staining. Representative results from **(M)** solvent- (water), **(N)** UV-irradiated- (positive control), and **(O,P)** BAY-61-3606-treated activated PMNs.

To further validate the contribution of SYK to IC-induced neutrophil activation, we stimulated neutrophils or PMNs with IgG IC in presence of different concentrations of different SYK inhibitors (BAY61-3606 or PRT062607). BAY61-3606 dose-dependently reduced IgG IC-triggered release of ROS from PMNs (Figures 6A,B). Consistent with this finding, PRT062607 significantly reduced IgG IC-triggered ROS (not shown). In some PD, i.e., EBA, anti-COL7 IgA is the only identified Ig class in approximately 30% of patients (42). Furthermore, anti-COL7 IgA induces PMN activation and subepidermal blistering *in vitro* (27, 43). We thus assessed whether SYK blockade can also modulate ROS release from IgA IC-activated PMNs. Compared to IgG-IC-activated PMNs, we observed an almost identical level of inhibition (Figures 6C,D). In line, BAY61-3606 also ablates ROS-dependent dermal–epidermal separation in cryosections of human skin incubated with anti-COL7 IgG and PMNs (Figures 6E–H). Regarding other neutrophil responses, inhibition of SYK normalized activation-triggered CD66b expression, but had no effect on L-selectin sheading (Figures 6I–L). These effects of BAY61-3606 were achieved at non-toxic concentrations (Figures 6M–P).

### BAY61-3606 Ablates Signaling Events in IC-Activated PMN

**Figure 7:**
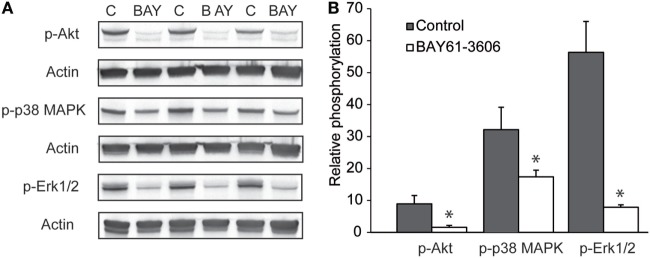
Pharmacological inhibition of SYK ablates signaling events in immune complex-activated polymorphonuclear leukocyte (PMN). **(A)** Representative blots from immune complex-activated PMN (“C”) or activated PMNs treated with BAY61-3606 (“BAY”) from three blood donors. **(B)** Image analysis showed that BAY61-3606 ablated pAkt phosphorylation and significantly reduced p38 and Erk phosphorylation. The data are presented as the mean (boxes) and STD (error bars) and are based on three experiments per group (*p < 0.05, *t*-test).

**Figure 8:**
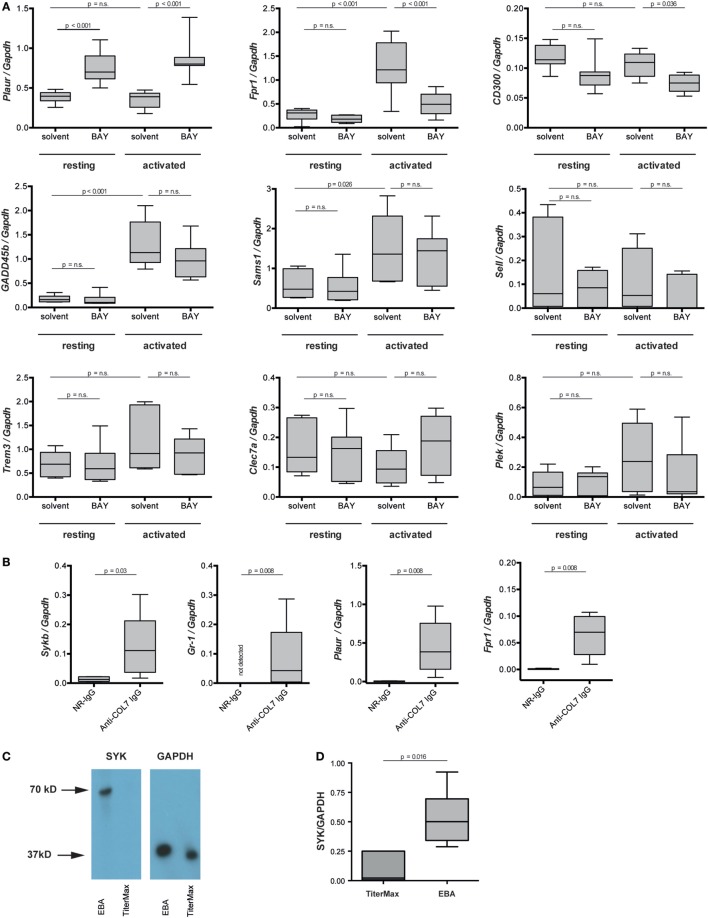
*Plaur* and formyl peptide receptor 1 (*Fpr1*) are regulated by SYK in immune complex-activated murine neutrophils. **(A)** Neutrophils were activated by immune complexes in absence or presence of BAY-61-3606 (BAY). Solvent and resting cells served as controls. Plots represent the expression of indicated gene in relation to *Gapdh*. Because of the non-parametric distribution, data are presented as median (centered vertical line), 25/75-percentile (boxes), and the 5/95-percentile (bars). Data are based on 8–10 samples per group. Statistical analysis was performed using ANOVA Ranks with the Student–Newman–Keuls post-test. **(B)** mRNA expression of *Sykb, Gr-1, Plaur*, and *Fpr1* were determined in the skin of healthy mice (NR-IgG) and mice with experimental epidermolysis bullosa acquisita (EBA) (anti-type VII collagen IgG). For expression analysis, skin specimens from corresponding areas were obtained. Myeloid cell infiltration, mirrored by an increase in *Gr-1* expression, was accompanied by an increased expression of *Sykp, Plaur*, and *Fpr1*. Data are based on five mice per group. Statistical calculations were performed using Rank Sum test. **(C)** Western blot analysis of SYK and GAPDH expression in the same samples of immunization-induced EBA and normal mouse skin. SYK expression could, in most cases, be detected only in lesional skin—with very few SYK expression in healthy skin. The graph shows the relative amount of the mean gray value (MD) of the SYK bands per GAPDH bands. Here representative blots are shown. **(D)** Quantitative analysis of the Western blots from five mice per group (*t*-test).

Regarding the mechanisms downstream of SYK, we extended our previous findings that the IC-induced activation of PMNs leads to the phosphorylation of ERK, AKT, and p38 (11). By adding BAY61-3606 to IC-activated PMNs, we show that SYK blockade leads to a reduction in the phosphorylation of ERK, AKT, and p38 in IC-activated PMNs (Figure 7). We next aimed to obtain novel insights into the pathways controlled by SYK. For this, PMNs were activated with ICs in the presence or absence of BAY61-3606, and expression levels of nine randomly genes from the predicted gene network controlled by SYK (Figure 1B) were evaluated. Two novel SYK-interacting genes, *Plaur* and *Fpr1*, were validated (Figure 8A). The latter is downregulated after the inhibition of SYK in IC-activated PMNs, while *Plaur* expression increased if SYK was inhibited. Furthermore, within the predicted co-expression network, several interacting partners of SKY were validated by curated databases (not shown); examples include *Plek, Ppbp, Clec7a*, and *Nlrp3*, which were predicted by the STRING database. Furthermore, the expression of *Sykb, Plaur*, and *Fpr1* were determined in skin specimen from mice with and without experimental EBA. In line with the data shown in Figure 1, an increased expression of *Sykb* was noted in mice with experimental EBA. Similar differences among healthy and disease mice were observed for *Plaur* and *Fpr1* (Figure 8B). RT-PCR data were confirmed using western blotting, detecting SYK (Figures 8C,D) almost exclusively in skin affected by EBA skin lesions.

## Discussion

In an unbiased approach using whole-genome expression profiling, aiming to pinpoint novel therapeutic targets for the treatment of EBA and other PD, we identified *Sykb* as a hub gene in mice with experimental EBA. Based on this morphological observation, we hypothesized that inhibition of SYK may have either anti- or pro-inflammatory effects. In support of the first assumption, SYK acts downstream of activating FcγR (44), which is essential to mediate inflammation in PD (9, 45) and other autoantibody-mediated diseases (46). By contrast, we recently identified an inhibitory signaling cascade, triggered by binding of highly galactosylated ICs to FcγRIIB and dectin-1 to block the pro-inflammatory signaling triggered by C5aR1. Inhibition of signaling downstream of C5aR1 was mediated by tyrosine phosphorylation of the ITAM-like motif downstream of dectin-1 and transient phosphorylation of SYK (6). Our results clearly document that SYK expression by myeloid, but not lymphoid, cells is an absolute requirement for induction of inflammation in antibody transfer-induced EBA, which has also been recently demonstrated elsewhere (47). In this paper, the authors also demonstrated a complete lack of skin lesion in SKY-deficient mice, which were generated by injection of bone marrow cells of *Syk*^tm1Tyb^ mice into lethally irradiated CD45.1^+^ recipient wild-type mice. Hence, also herein, SKY expression on radiosensitive (hematopoietic) cells has convincingly been demonstrated. By use of myeloid- and lymphoid cell-specific SYK-deficient mice, we are here able to link the SYK-dependency to the myeloid cell lineage (Figures 4 and 5). This is in line with the *in vitro* observations made here (Figure 6) and by Németh et al. who also demonstrated a complete unresponsiveness of neutrophils from SYK-deficient mice to stimulation with ICs (47). In experimental EBA, myeloid and T cells have been demonstrated to mediate skin inflammation and blistering (7, 48), while mast cells were activated, but not required for clinical disease induction (49). Based on the findings presented here, myeloid, but not T cell expressed SYK mediates EBA pathogenesis. Of these, both neutrophils and monocytes/macrophages, which have been recently been demonstrated to contribute to EBA pathogenesis (50), are the two most likely cell types expressing SYK and contributing to EBA pathogenesis.

The almost complete absence of dermal myeloid cell infiltration after genetic or pharmacologic SYK inhibition is, at first glance, puzzling, because ablation of Syk does not greatly impair the migration properties of neutrophils (51). Furthermore, similar observations were made in mice lacking FcγR IV expression (9). We therefore hypothesize that when myeloid cells become activated by ICs located at the DEJ, they not only mediate blistering, but also release numerous mediators that in turn trigger an amplification of myeloid cell recruitment into the skin. So far, this has not been formerly demonstrated for EBA. In experimental BP, neutrophil elastase degrades collagens, leading to the formation of chemotactic peptides, which sustains the influx of neutrophils into the skin (52).

We next aimed to validate the predicted *Sykb*-gene network (Figure 1B). Within the predicted co-expression network, several interacting partners of SKY (e.g., *Cd300b, Tlr13, Jdp2*, and *Nfkbid*) were validated by curated databases (not shown). In addition, two novel *Sykb*-interacting genes, *Plaur* and *Fpr1*, were validated. *Plaur* (plasminogen activator, urokinase receptor) is expressed in conjunction with the C3-receptor on the surface of neutrophils (53). The function of *Plaur* has been linked to neutrophil migration (54, 55). *Fpr1* is expressed on activated neutrophils and promotes further activation upon ligand binding (56).

**Figure 9:**
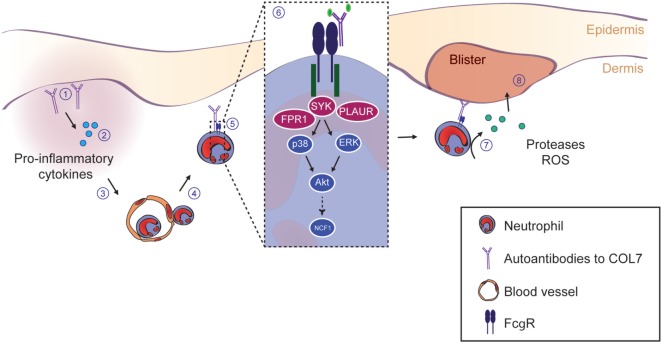
SYK and downstream kinases are essential to drive inflammation and blistering in experimental epidermolysis bullosa acquisita (EBA). This schematic summarizes the current understanding of the events leading to blistering in experimental EBA. (1) The initial event is the binding of the IgG and/or IgA autoantibodies directed against type VII collagen (COL7). (2) Thereafter, anaphylatoxins are generated by activation of the complement cascade. Furthermore, several cytokines lipid mediators are released, which collectedly leads (3) to the activation of endothelial cells and allows the (4) CD18/ICAM-1-dependent extravasation of Gr-1^+^ myeloid cells into the skin. (5) Within the skin, myeloid cells bind to the skin-bound immune complexes *via* specific activating Fc gamma receptors (FcγR). (6) FcγR binding triggers an intracellular signaling cascade involving SYK, p38, ERK, and Akt, ultimately leading to the activation of the NCF1 gene, which is part of the NADPH oxidase complex and generates reactive oxygen species (ROS). Within this pathway, we show an absolute requirement of SYK for blister induction in EBA. Blockade of downstream kinases (i.e., p38, Akt, or individual Src family kinases) only partially reduces the blistering phenotype, indicating that SYK activation is in the center stage of EBA pathogenesis. (7) Ultimately, this intracellular signaling process leads to the release of ROS and proteases from the myeloid cells, which (8) mediates blistering. Image modified from Ludwig et al. (2).

Our finding adds to the current view of mechanisms leading to tissue damage in EBA (2), which is initiated and triggered by the binding of IgG/A autoantibodies to COL7. This initial binding leads to increased concentrations of potent chemoattractants such as C5a (57) and leukotriene B4 (58), which lead to a CD18/ICAM-1-dependent influx of myeloid cells into the skin (7, 8). In the skin, myeloid cells bind to the tissue-deposited ICs in an FcγRIV-dependent fashion (9). This engagement of FcγR to ICs triggers intracellular signaling, involving SYK, PI3Kβ and δ, AKT, p38 MAPK, ERK, Src family kinases, CARD9, and RORα (10–14, Koga et al., submitted^1^). Of note, we here demonstrate that ERK, AKT, and p38 act downstream of SYK, which is (at least) required for ROS release and degranulation (Figure 6). The two newly identified interacting genes of *Sykb*, namely *Fpr1* and *Plaur*, most likely also act downstream of *Sykb*, but this awaits experimental confirmation. Collectively, myeloid cell activation leads to cytokine release, which sustains further neutrophil recruitment (8, 50, 59) and, through release of ROS and proteases, induces subepidermal blistering (7, 16). This concept of autoantibody-mediated tissue damage in EBA is graphically summarized in Figure 9.

Collectively, these insights into EBA pathogenesis were driven by an unbiased expression profiling approach and identified myeloid SYK as a central player in driving inflammation in a prototypical autoantibody-induced disease. Furthermore, we recognized and experimentally validated novel gene interaction partners of SYK, specifically *Fpr1* and *Plaur*. This should encourage the exploitation of SYK and SYK-regulated genes as potential therapeutic targets for EBA, as well as diseases with autoantibody-driven pathology.

## Ethics Statement

Foreskin and blood collections from healthy volunteers and patients were performed after written informed consent was obtained. All experiments with human samples were approved by the ethical committee of the Medical Faculty of the University of Lübeck (reference numbers: 09-140, 04-061, 04-144, 05-056) and were performed in accordance with the Declaration of Helsinki.

## Author Contributions

US, NM, AK, KB, ES, TL, AR, SG, GV, FS, MA, KD, HP, MJ, KK, DZ, and SI performed experiments, YG performed statistical analysis, US and RL designed the study. All authors critically evaluated the data, wrote the manuscript, and approved the final version for publication.

## Conflict of Interest Statement

The authors declare that the research was conducted in the absence of any commercial or financial relationships that could be construed as a potential conflict of interest.

## Footnotes

^1^Koga H, Kasprick A, Lopez R, et al. Therapeutic effect of a novel PI3Kδ inhibitor in experimental epidermolysis bullosa acquisita. *Front Immunol* (2017).
