# Supplementary material for: Whole-Genome Expression Profiling in Skin Reveals SYK As a Key Regulator of Inflammation in Experimental Epidermolysis Bullosa Acquisita
Source: Front Immunol. 2018 Feb 15;9:249. doi: 10.3389/fimmu.2018.00249 (PMC5818881; doi:10.3389/fimmu.2018.00249)
Supplement: Supplementary file 1 [file Table_1.PDF]

Supplement Table 1. Top 20 differentially expressed genes between mice without clinical manifest EBA and healthy mice.

ID	Gene Name	log FC	*p-value*	*Adjusted p-value*
10380419	Col1a1	1.092457892	1.71E-11	2.97E-07
10529457	Cpz	0.476105771	1.83E-11	2.97E-07
10595211	Col12a1	0.937846131	3.26E-11	2.97E-07
10346015	Col3a1	0.986338829	3.42E-11	2.97E-07
10531724	Plac8	1.014783794	1.21E-10	8.43E-07
10583056	Mmp12	0.831790171	2.46E-10	1.20E-06
10536220	Col1a2	0.933247962	2.64E-10	1.20E-06
10460782	Gpha2	-0.588016109	2.77E-10	1.20E-06
10560919	Atp1a3	0.485810749	3.51E-10	1.36E-06
10560685	Bcl3	0.577733423	7.41E-10	2.58E-06
10354309	Col5a2	0.496367606	1.15E-09	3.39E-06
10546450	Adamts9	0.501134973	1.17E-09	3.39E-06
10556082	Ppfibp2	-0.28734775	1.29E-09	3.44E-06
10379636	Slfn4	1.289214032	1.60E-09	3.96E-06
10572949	Nr3c2	-0.319316147	1.90E-09	4.41E-06
10367400	Mmp19	0.798947908	2.12E-09	4.62E-06
10352143	Kif26b	0.383798765	2.39E-09	4.75E-06
10557895	Itgax	0.490973272	2.46E-09	4.75E-06
10403743	Inhba	0.618346825	3.67E-09	6.63E-06

ID corresponds to Affymetrix ID, Gene name is the official gene symbol, positive log FC show up-regulation and negative log FC shows down-regulation. The adjusted p-values have been corrected for multiple testing using Bonferroni correction. A list of all differently expressed genes is given in supplement table 2.
